# Purification and characterization of human adipose-resident microvascular endothelial progenitor cells

**DOI:** 10.1038/s41598-022-05760-4

**Published:** 2022-02-02

**Authors:** Natsumi Saito, Takako Shirado, Hitomi Funabashi-Eto, Yunyan Wu, Masanori Mori, Rintaro Asahi, Kotaro Yoshimura

**Affiliations:** 1 Department of Plastic Surgery, Jichi Medical University, 3311-1, Yakushiji, Shimotsuke, Tochigi 329-0498 Japan; 2 Department of Plastic Surgery, Federation of National Public Service Personnel Mutual Aid Associations, Hamanomachi Hospital, 3-3-1, Nagahama, Chuou-ku, Fukuoka, 810-8539 Japan

**Keywords:** Isolation, separation and purification, Regenerative medicine, Stem-cell biotechnology, Tissue engineering, Adult stem cells, Skin stem cells, Angiogenesis

## Abstract

Human adipose tissue is a rich source of adipose-derived stem cells (ASCs) and vascular endothelial progenitor cells (EPCs). However, no standardized method has been established for the isolation and purification of adipose-derived EPCs (AEPCs). The aim of this study was to establish a method for the isolation and purification of AEPCs. The stromal vascular fraction (SVF) was extracted from human lipoaspirates, and the CD45^−^CD31^+^ fraction of the SVF was collected by magnetic-activated cell sorting (MACS). The CD45^−^CD31^+^ fraction was cultured for 4.5 days, followed by a second MACS separation to collect the CD31^+^ fraction. Purified AEPCs were expanded without being overwhelmed by proliferating ASCs, indicating that a high level (> 95%) of AEPC purification is a key factor for their successful isolation and expansion. AEPCs exhibited typical endothelial markers, including CD31, von Willebrand factor, and the isolectin-B4 binding capacity. AEPCs formed colonies, comparable to cultured human umbilical vein endothelial cells (HUVECs). Both AEPCs and HUVECs formed capillary-like networks in the tube formation assay, with no significant difference in network lengths. We are the first to establish a purification and expansion method to isolate these cells. Because adipose tissue is a clinically accessible and abundant tissue, AEPCs may have potential advantages as a therapeutic tool for regenerative medicine.

## Introduction

Human endothelial progenitor cells (EPCs) were first identified in the circulating peripheral blood of adults^1^ and were later found in the bone marrow and umbilical cord blood^2–4^. EPCs recruited from the bone marrow are mobilized by the circulating blood and delivered to damaged organs, where they are incorporated into the vasculogenesis process and release multiple cytokines^1,5–7^. In addition to bone marrow-derived EPCs, EPCs localized in the blood vessel wall and microvasculature in various adult tissues, known as tissue-resident EPCs, contribute to homeostasis and regeneration after local injuries^8–11^. Tissue-resident EPCs have clonogenic and proliferative potential and express endothelial cell (EC)-specific markers^12,13^, including CD31, CD157^9,11^, c-kit^8^, and protein C receptor^14^, which are expressed in an organ-dependent manner. EPCs play pivotal roles in angiogenesis and vasculogenesis in various ischemic diseases^15–20^ and are expected to be potential therapeutic tools in regenerative medicine.

Human adipose tissue contains abundant mesenchymal stem cells, known as adipose-derived stem cells (ASCs), which have previously been used in clinical trials and therapies to treat ischemic, inflammatory, and degenerative diseases^21^. Adipose tissue represents an accessible and abundant cell source in humans, rich in capillary networks directly attached to every adipocyte. Liposuction aspirates can be enzymatically digested to obtain a stromal vascular fraction (SVF) that contains ASCs, resident macrophages, lymphocytes, and adipose-resident microvascular EPCs (AEPCs)^22,23^. During the adherent expansion of SVFs in culture, ASCs can easily be expanded, but AEPCs are driven away by proliferating ASCs over time. Although human AEPCs are expected to serve as a promising tool in regenerative medicine to treat ischemic and degenerative diseases due to its physiological functions, no standardized method has yet been established to purify and expand AEPCs. Thus, human AEPCs have not yet been clinically tested.

The aim of this study was to establish a method for purifying and expanding human functional AEPCs for future therapies.

## Materials and methods

### Ethics approval and consent to human participants

All the experimental protocols involving human subjects were approved by the institutional review board (IRB) of Jichi Medical University Hospital (approval number: I17-049). All the participants provided signed informed consent before their inclusion in the study. No personal details or contact data of any patient have been included. All the experiments were performed in accordance with relevant guidelines and regulations.

### Extraction and cultivation of the SVF

After obtaining informed consent, we used the IRB-approved protocol to collect human lipoaspirates from 17 healthy donors aged 28–66 years who had undergone liposuction of the abdomen or thighs.

**Figure 1:**
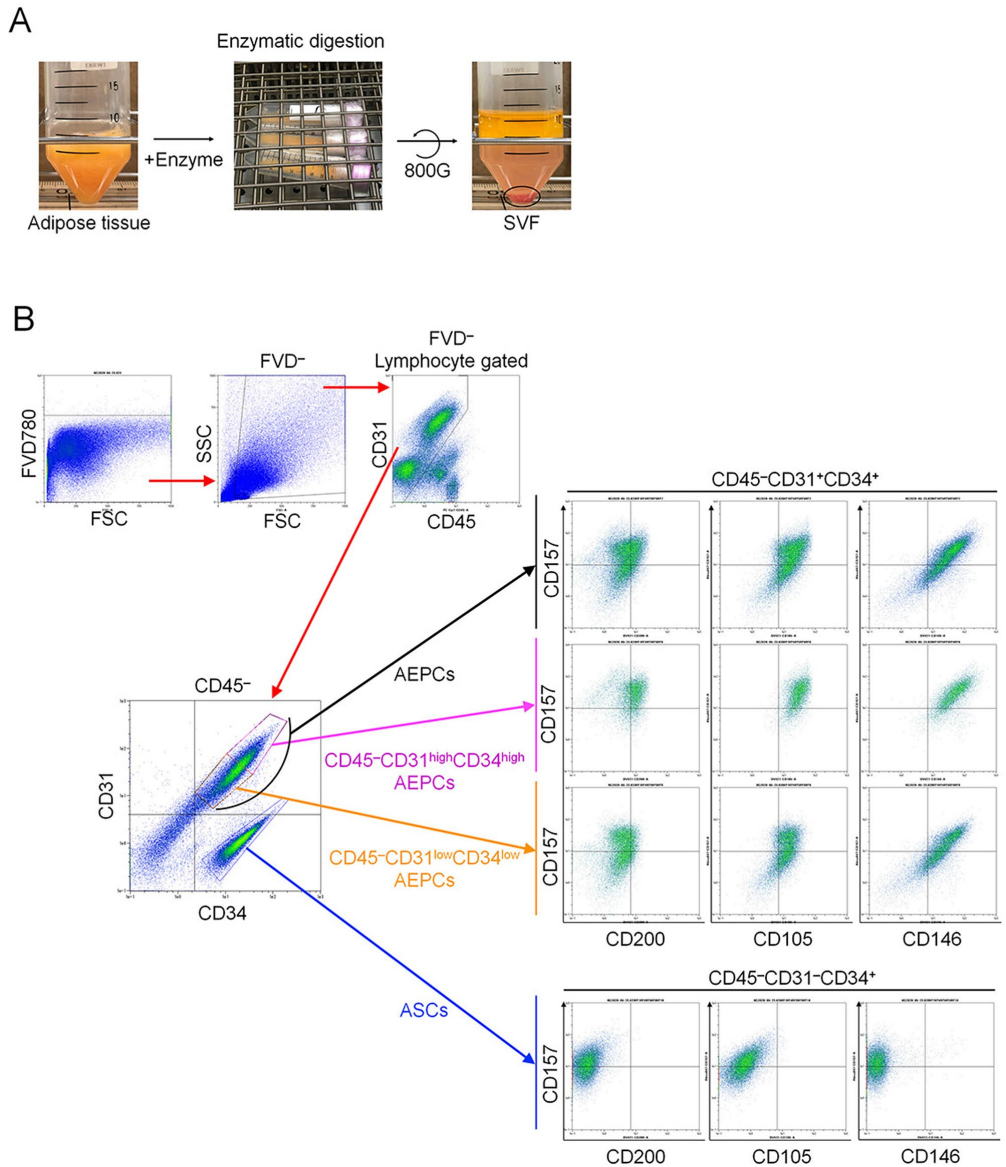
SVF extraction and characterization. (**A**) The SVF was enzymatically isolated from human lipoaspirates using a collagenase-based enzymatic solution at 37 °C for 30 min at 120 rpm of reciprocating motion, followed by centrifugation at 800×*g* for 10 min. The open circle indicates the SVF pellet. (**B**) The freshly isolated SVF was characterized by the expression of CD45, CD34, CD31, CD105, CD146, CD157, and CD200 during the flow cytometry analysis. The experiment was performed independently 3 times (3 donors).

After natural gravity sedimentation, lipoaspirates develop three layers: oil, adipose tissue, and tumescent liquid. The floating adipose tissue layer was extracted, measured, and digested with an equivalent volume of collagenase-based enzyme solution (details below) at 37 °C for 30 min at 120 rpm of reciprocating motion (Yamato Scientific), followed by centrifugation at 800×*g* for 10 min. The resulting cell pellet was designated the SVF (Fig. 1A). After washing with Hanks’ Balanced Salt Solution (HBSS; Thermo Fisher Scientific; #14175-103), the SVF was sequentially passed through 100-µm and 40-µm sieve cell strainers (Corning). The strained cell suspension was centrifuged again at 800×*g* for 5 min at 4 °C, and the obtained SVF pellet was washed with HBSS. The nucleated cell number, viability, and total cell-sized particle number were measured using a fluorescent cell counter (LUNA-STEM; Logos Biosystems) after double staining with acridine orange and propidium iodide (Logos Biosystems).

We used flow cytometry to examine the extraction efficiency of AEPCs using three collagenase-based enzyme formulations: (#1) 0.2% (w/v) collagenase (crude type; FUJIFILM Wako Pure Chemical; #032-22364) and 3 mM CaCl_2_ (FUJIFILM Wako; #037-24031) in HBSS; (#2) 0.2% (w/v) collagenase, 3 mM CaCl_2_, and 1000 U/mL of deoxyribonuclease 1 (DNase1; Worthington Biochemical; #LS002139) in HBSS; (#3) 0.2% (w/v) collagenase, 3 mM CaCl_2_, 1000 U/mL DNase1, and 0.1% (v/v) Poloxamer 188 solution (Pol188; Sigma-Aldrich) in HBSS. Pol188 has previously been reported as an additive to elevate the extraction efficiency of ASCs in the SVF^24^.

### Flow cytometry analysis

Before flow cytometry of the SVF, hemolysis was performed using red blood cell lysis solution (Miltenyi Biotec), according to the manufacturer’s instructions. Hemolyzed cells were resuspended in fluorescence-activated cell sorting (FACS) buffer, comprising 0.5% (w/v) bovine serum albumin (BSA; Sigma-Aldrich; #A8806) and 2 mM ethylenediamine-*N*,*N*,*N*′,*N*′-tetraacetic acid (EDTA; DOJINDO; #N001) in Dulbecco’s phosphate-buffered saline (DPBS; Thermo Fisher Scientific; #14190250).

Cultured cells were dissociated with TrypLE Express Enzyme (Thermo Fisher Scientific) for 5 min at 37 °C in a CO_2_ incubator. The enzymatic reaction was stopped by adding cold medium containing fetal bovine serum (FBS), and the cells were resuspended in FACS buffer.

Cell samples were analyzed with a flow cytometer (Miltenyi Biotec MACSQuant Analyzer, and MACSQuantify software ver. 2.5) for the cell size, granularity, and expression of CD45, CD31, CD34, CD105, CD146, CD157, and CD200. Viable cells were selected using a live/dead cell staining dye (Fixable Viability Dye eFluor 780, FVD; Thermo Fisher Scientific). After treatment with Fc receptor blocker (Human BD Fc Block Reagent; BD Pharmingen; #564220) to block nonspecific binding, the cells were reacted with fluorescent-conjugated specific antibodies for 30 min on ice, washed, and resuspended in FACS buffer. Both an isotype control and a universal negative control were used to designate the negative population in all channels. The voltage conditions and compensation settings were determined for the negative controls and single-fluorescent-staining samples. The antibodies used are shown in Table 1.

**Table 1:** List of antibodies used during flow cytometric analysis.

Antigen	Labeling	Clone name	Supplier, product #	Isotype control
CD45	APC	HI30	BD #555485	BD #550854
CD45	PE-Cy7	HI30	BD #557748	BD #557872
CD34	FITC	581/CD34	BD #555821	BD #555748
CD31	PE	WM59	BD #555446	BD #555749
CD146	BV421	P1H12	BD #564325	BD #562438
CD105	BV421	266	BD #563920	BD #562438
CD157	Alexa Fluor 647	SY/11B5	BD #564870	BD #557714
CD200	BV421	MRC OX-104	BD #564114	BD #562438

### Purification of AEPCs

The hemolyzed SVF was applied to a magnetic-activated cell sorting (MACS) system using CD45 and CD31 microbeads (Miltenyi Biotec; #130-045-801 and #130-091-935) consecutively, according to the manufacturer’s instructions. The separation buffer (MACS buffer) consisted of 0.5% (w/v) BSA (Sigma-Aldrich; #A8806) and 2 mM EDTA in HBSS. Both the negative and positive fractions separated using MACS MS columns (Miltenyi Biotec) were collected. CD45^−^CD31^+^ (AEPC-enriched fraction) and CD45^−^CD31^−^ (ASC-enriched fraction) fractions were seeded on cell culture plates (Corning) at 1 × 10^4^ cells/cm^2^ in cell culture medium (EGM-2MV BulletKit; Lonza; #CC-3202) (Supplemental Table S1) at 37 °C in 5% CO_2_ and 95% air. A second MACS procedure using only CD31 microbeads was later applied to further purify AEPCs.

### Immunocytochemistry

AEPCs and human umbilical vein endothelial cells (HUVECs; Lonza; #C2519AS) were seeded at 2.5 × 10^3^ cells/cm^2^ and cultured in EGM-2MV and EGM-2 on glass-based chamber slides (AGC TECHNO GLASS), respectively. The cells were fixed with 4% paraformaldehyde in phosphate buffer solution (PFA; FUJIFILM Wako), washed three times with 10 mM glycine in phosphate buffered saline (PBS; 137 mM NaCl, 2.7 mM KCl, 10 mM Na_2_HPO_4_, 1.8 mM KH_2_PO_4_ (all reagents from FUJIFILM Wako) in H_2_O), and permeabilized with 0.1% Triton X-100 (FUJIFILM Wako) in PBS for 5 min. The cells were then washed three times with PBS, blocked with 3% BSA in PBS for 10 min, and washed once with PBS. The specimens were then incubated overnight at 4 °C with primary antibodies, either mouse anti-human CD31/PECAM-1 (R&D Systems; #BBA7; clone #9G11; 10 µg/mL) or mouse anti-human von Willebrand factor (vWF; Thermo Fisher Scientific; #MA5-14029; clone #F8/86; 2 µg/mL) diluted in PBS containing 1% BSA. Purified normal mouse IgG1 (BD Biosciences; #550878; 10 µg/mL) was used as a negative control. The specimens were washed three times with 0.1% BSA in PBS and incubated with secondary antibodies Alexa Fluor 594 goat anti-mouse IgG1 (Thermo Fisher Scientific; #A21125; 1000-fold dilution) and Alexa Fluor 488-Isolectin GS-IB_4_ (Thermo Fisher Scientific; #I21411; 5 µg/mL) in PBS containing 1% BSA and 0.3 mM CaCl_2_ for 1 h at room temperature (RT). After washing three times with PBS containing 0.1% BSA and 0.3 mM CaCl_2_, the slides were mounted with aqueous mounting media containing 4′,6-diamidino-2-phenylindole (DAPI; VECTOR Laboratories). Microphotographs were obtained using a confocal microscope (OLYMPUS Life Science; #FV1000).

### Endothelial cell colony-forming unit assay

The EC colony-forming unit (CFU-EC) assay was modified from the fibroblastoid colony-forming unit assay^22^. Cultured AEPCs (*P* = 5) and HUVECs (*P* = 5) were dissociated using TrypLE Express Enzyme. The cells were seeded on cell culture plates (Corning; #353502) at 125 cells per 35-mm-diameter well (12.6 cells/cm^2^) and cultured for 7 days. To visualize colonies, the cells were fixed with 4% PFA in PBS for 15 min at RT, stained with 0.05% (w/v) crystal violet (FUJIFILM Wako) for 30 min at RT, and washed with distilled water. Serial photographs were obtained using a light field microscope (Keyence; #BZ-X710), and the digitized images were combined to generate a single large image using analyzer software (Keyence).

### Endothelial cell network formation assay

Expanded AEPCs and HUVECs (50 µL 2 × 10^5^ cells/mL of each complete medium) were plated on a 96-well plate (PerkinElmer) precoated with 50 µL sol–gel transited Matrigel (Corning; #356237) and incubated for 6 h at 37 °C in a CO_2_ incubator. Photographs were obtained using an inverted phase-contrast microscope with a camera (Leica Microsystems; DM IL LED and MC170 HD). Images were analyzed using the Angiogenesis Analyzer plug-in for ImageJ software^25^ according to the standard protocol found on the developer’s website. Although this software can analyze various parameters in constitutive elements of tubular networks, we measured the total segment length, total branch length, and total length as appropriate evaluation standards. Because AEPCs showed stronger cell–cell interactions than HUVECs during the cell dissociation step, the AEPC suspension consisted of both single cells and cell clusters containing 2–4 cells.

### Statistical analysis

Quantitative data were presented as means ± standard deviation (SD). We used two-tailed unpaired *t*-test for the following analyses: immunocytochemistry, CFU-EC assay, and EC network formation assay. All statistical analyses were performed using Prism 6 ver. 6.0d (GraphPad Software).

## Results

### Flow cytometric analysis of the SVF

Freshly isolated SVF samples contained 3.84 ± 1.43 × 10^5^ cells per gram of adipose tissue (n = 9). The SVF could be segregated into four primary cell populations, AEPCs (CD45^−^CD34^+^CD31^+^), ASCs (CD45^−^CD34^+^CD31^−^), hematopoietic cells (CD45^+^), and other cell types (CD45^−^CD34^−^; Fig. 1B). The proportions of hematopoietic cells, AEPCs, ASCs, and other cells in the SVF were 41.1 ± 10.0%, 25.2 ± 6.8%, 21.0 ± 2.8%, and 12.7 ± 4.7%, respectively. Fresh AEPC population was further analyzed for other surface markers and characterized as CD45^−^CD31^+^CD34^+^CD105^+^CD146^+^CD157^±^CD200^±^. The AEPC population could be further divided into two subpopulations, CD31^high^CD34^high^ and CD31^low^CD34^low^. The CD31^high^CD34^high^ AEPC population showed high expression levels of CD157 and CD200, whereas the CD31^low^CD34^low^ AEPC population presented various fluorescent intensities for CD157 and CD200 expression (Fig. 1B). Fresh ASC population was characterized as CD45^−^CD31^−^CD34^+^CD105^−^CD146^−^CD157^±^CD200^−^. CD105 is a well-known mesenchymal stem cell marker, and ASCs expressed CD105 in vitro after adhering to the plastic surfaces of culture plates (Fig. 4).

### Purification and expansion of AEPCs

As a preliminary experiment, we attempted to extract the SVF using three enzyme solutions: #1: collagenase and CaCl_2_; #2: collagenase, CaCl_2_, and DNase1; #3: collagenase, CaCl_2_, DNase1, and Pol188. The percentage of AEPCs in the SVF tended to be higher when extracted using enzyme solution #2 (Supplemental Fig. S1A). We also examined the effects of various collagenase concentrations (0.0–2.0%) in enzyme solution #2. The numbers of total nucleated cells and ASCs in the SVF increased in a dose-dependent manner with increasing collagenase concentrations. The numbers of AEPCs in the SVF peaked at collagenase concentrations of 0.1–0.4% (Supplemental Fig. S1B). Based on these results, the same volume of the #2 enzyme formulation containing 0.2% (w/v) collagenase, 3 mM CaCl_2_, 1000 U/mL DNase1, and HBSS was added to adipose tissue in subsequent experiments.

We also examined the use of several good manufacturing practice (GMP)-grade enzymes and combinations to prepare purified AEPCs for clinical use (Supplemental Fig. S1C).

**Figure 2:**
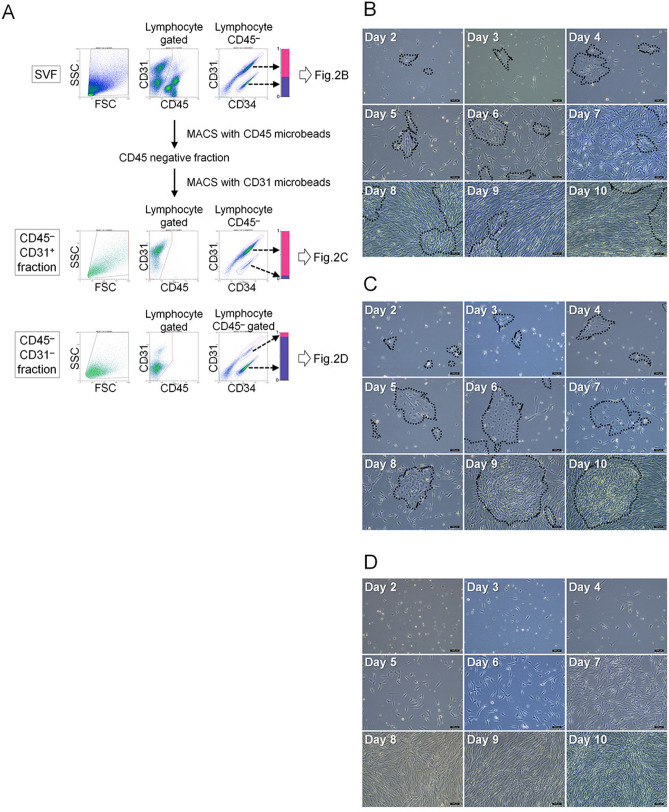
MACS separation and culture of the SVF. (**A**) The freshly isolated, hemolyzed SVF was separated into CD45^−^CD31^+^ (AEPC-rich) and CD45^−^CD31^−^ fractions (ASC-rich) in the first MACS separation. During this flow cytometric analysis, lymphocyte gating events in the SSC versus SFC plot were set to 100%. CD45^−^CD34^+^CD31^+^ (magenta ROI) was defined as the AEPC population. CD45^−^CD34^+^CD31^−^ (violet ROI) was defined as the ASC population. Bar charts display the ratio between the CD45^−^CD31^+^CD34^+^ and CD45^−^CD31^+^CD34^−^ populations in the SVF (upper panels), CD45^−^CD31^+^, (middle panels), and CD45^−^CD31^−^ fractions (lower panels), which were 0.54 to 0.46 ± 0.075 (n = 9, 9 donors, mean ± SD), 0.95 to 0.05 ± 0.03 (n = 5, 5 donors, mean ± SD), and 0.18 to 0.82 ± 0.08 (n = 5, 5 donors, mean ± SD), respectively. Cultivation of the (**B**) freshly isolated SVF, (**C**) first MACS-separated CD45^−^CD31^+^ fraction (AEPC-rich), and (**D**) first MACS-separated CD45^−^CD31^−^ fraction (ASC-rich). The morphologies of all the populations were photographed daily from days 2–10 using a phase-contrast microscope (Leica DM IL LED with a camera MC170HD; 100× magnification). Bars represent 100 µm. All the cell populations were seeded at 1 × 10^4^ nucleated cells/0.2 mL of EGM-2 media/cm^2^. Dotted circles indicate AEPC colonies. The experiment was performed twice, independently from 2 donors.

The SVF was separated by MACS using CD45 and CD31 microbeads, both CD45^−^CD31^+^ (AEPC-rich) and CD45^−^CD31^−^ (ASC-rich) MACS-separated fractions were obtained, and the expression profiles of CD45, CD34, and CD31 were analyzed by flow cytometry (Fig. 2A). Population analysis showed that the CD45^−^CD31^+^ (AEPC-rich) fraction contained 84.3 ± 5.8% of AEPCs (CD45^−^CD34^+^CD31^+^ cells), leukocytes (CD45^+^ cells) at 0.9 ± 0.3%, ASCs (CD45^−^CD34^+^CD31^−^ cells) at 4.7 ± 2.3%, and others (CD45^−^CD34^−^ cells and debris) at 10.1 ± 5.0% (n = 5) (Fig. 2A). The SVF, CD45^−^CD31^+^ fraction, and CD45^−^CD31^−^ fraction were cultured for 10 days in EGM-2 media, and photographs were obtained daily from days 2 to 10 using a phase-contrast microscope, with representative photographs shown in Fig. 2B (SVF), Fig. 2C (CD45^−^CD31^+^ fraction) and 2D (CD45^−^CD31^−^ fraction). AEPCs (cobblestone shape) and ASCs (spindle shape) could be morphologically discriminated under the microscope. In AEPC-rich culture (CD45^−^CD31^+^ fraction), AEPC colonies (dotted line) were observed, and fewer ASCs were observed compared with the unenriched SVF culture until day 7 (Fig. 2B,C). However, even after enrichment, AEPC colonies were eventually overwhelmed by proliferating ASCs starting on day 10 (Fig. 2C), indicating that a single MACS enrichment step was insufficient for AEPC purification and expansion. Flow cytometric analyses indicated that the ASC proportion in the CD45^−^CD31^+^ (AEPC-rich) fraction increased after 5 days of culture (Supplemental Fig. S2).

**Figure 3:**
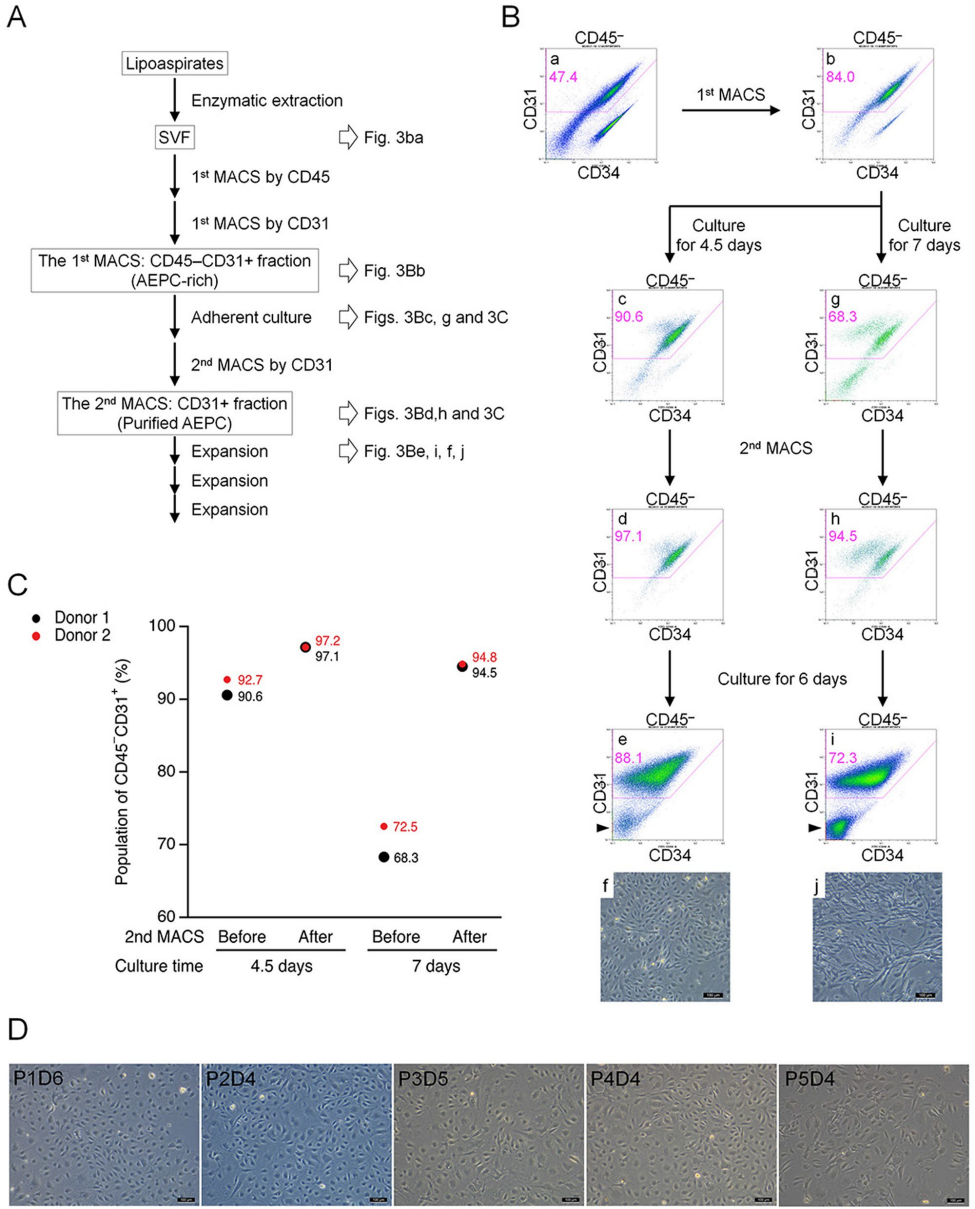
Establishment of the AEPC purification. (**A**) Schematic view of the AEPC purification method from human lipoaspirates. AEPCs were purified from the SVF using two MACS sorting steps (CD45^−^CD31^+^ sorting and only CD31^+^ sorting) and adherent cultures. (**B**,**C**) Optimization of the timing for the second MACS sorting step for AEPC purification. Cells were dissociated from the cell culture surface with TrypLE Express Enzyme after either 4.5 or 7 days of culture and were subjected to MACS separation using CD31 microbeads. Cell surface markers (CD45, CD31, and CD34) were examined by flow cytometry. The percentage of CD45^−^CD31^+^ (AEPC) cells in CD45^−^CD31^−^ (ASCs, isotype^+^ cells, other cells, and debris) cells is shown in magenta. ASCs drastically decreased CD34 expression in vitro, which could be observed in the CD45^−^CD31^−^ population (arrowheads). Scale bars in microscopic photographs represent 100 µm. This experiment was performed twice, independently from 2 donors. (**D**) Morphologies of purified AEPCs over time. The photographs were taken using a phase-contrast microscope (Leica DM IL LED with a camera MC170HD; 100× magnification). P1D6 indicates the culture period at passage 1, after 6 days in culture. The experiment was performed twice, independently from 2 donors. Bars represent 100 µm.

Therefore, we performed a second MACS enrichment using CD31 microbeads to further purify the AEPC population. A schematic view of the procedure of AEPC purification is shown in Fig. 3A. After performing purification steps of fresh SVF (Fig. 3Ba) and the first MACS CD45^−^CD31^+^ (AEPC-rich) fraction (Fig. 3Bb), the second MACS separation step of cultured AEPC-rich populations was performed after either 4.5 days (before the timing of ASC proportion increase) or 7 days (after the timing of ASC proportion increase) of culture (Fig. 3Bc,d,g,h). When separated on day 4.5 (steps in Fig. 3Bc,d), the respective percentages of CD45^−^CD31^+^ cells before and after the second MACS separation were 91.6% and 97.1%, respectively (n = 2; Fig. 3C). When separated on day 7 (steps in Fig. 3Bg,h), the respective percentages of CD45^−^CD31^+^ cells before and after separation were 70.4% and 94.7%, respectively (n = 2; Fig. 3C). AEPCs separated on day 4.5 were cultured for 6 days (Fig. 3Be,f) and expanded with satisfactory purity through at least 5 passages (Fig. 3D). By contrast, AEPCs separated on day 7 were overwhelmed by proliferating ASCs after 6 days of culture (Fig. 3Bi,j).

### Time-dependent changes in CD marker expression profiles AEPC and ASC cultures

**Figure 4:**
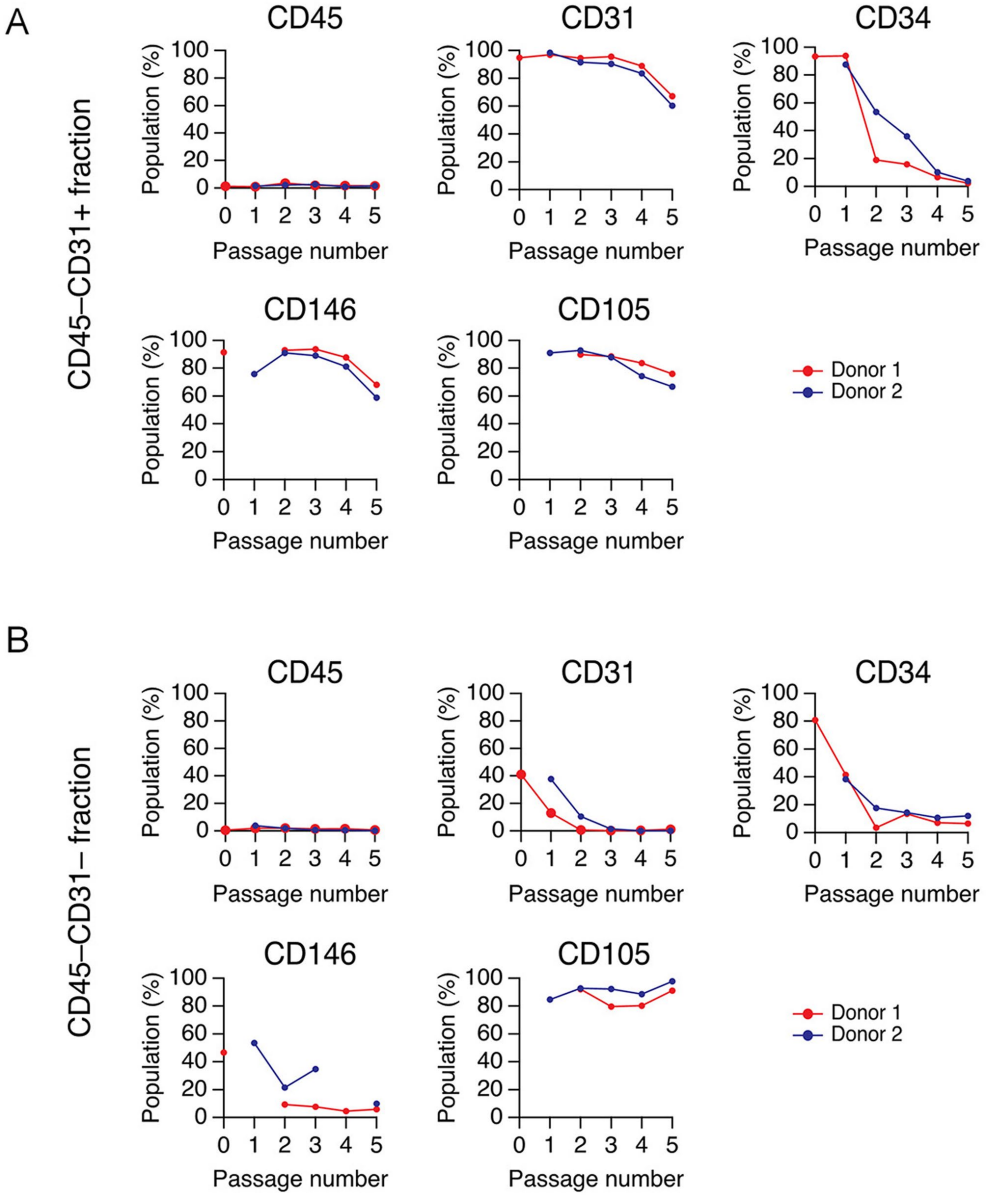
Time-dependent changes in AEPC cell morphologies and surface marker expression profiles. (**A**) The CD45^−^CD31^+^ and (**B**) CD45^−^CD31^−^ MACS-separated fractions were characterized for the expression of CD markers, including CD45, CD31, CD34, CD146, and CD105. The experiment was performed twice, independently from 2 donors. The graph indicates 2 donors, depicted by red and blue lines. The X-axis (passage number) indicates that the CD45^−^CD31^+^ fraction at P5 corresponded to dissociated AEPCs at P4D4, as shown in Fig. 3D.

AEPCs (obtained as the CD45^−^CD31^+^ fraction after two MACS separation steps) and ASCs (obtained as the CD45^−^CD31^−^ fraction after the first MACS separation) were cultured, and time-dependent changes in CD marker expression profiles (CD45, CD31, CD34, CD146, and CD105) were examined. Cultured AEPCs maintained CD31, CD146, and CD105 expression through at least 5 passages, whereas CD34 expression decreased over time in culture (Figs. 1B and 4). ASCs began to express CD105 after seeding, despite being CD105^−^ before seeding, and maintained expression through at least 5 passages, although CD34 expression decreased over time in culture. CD146 expression was not observed in ASCs.

### Characterization of expanded AEPCs

**Figure 5:**
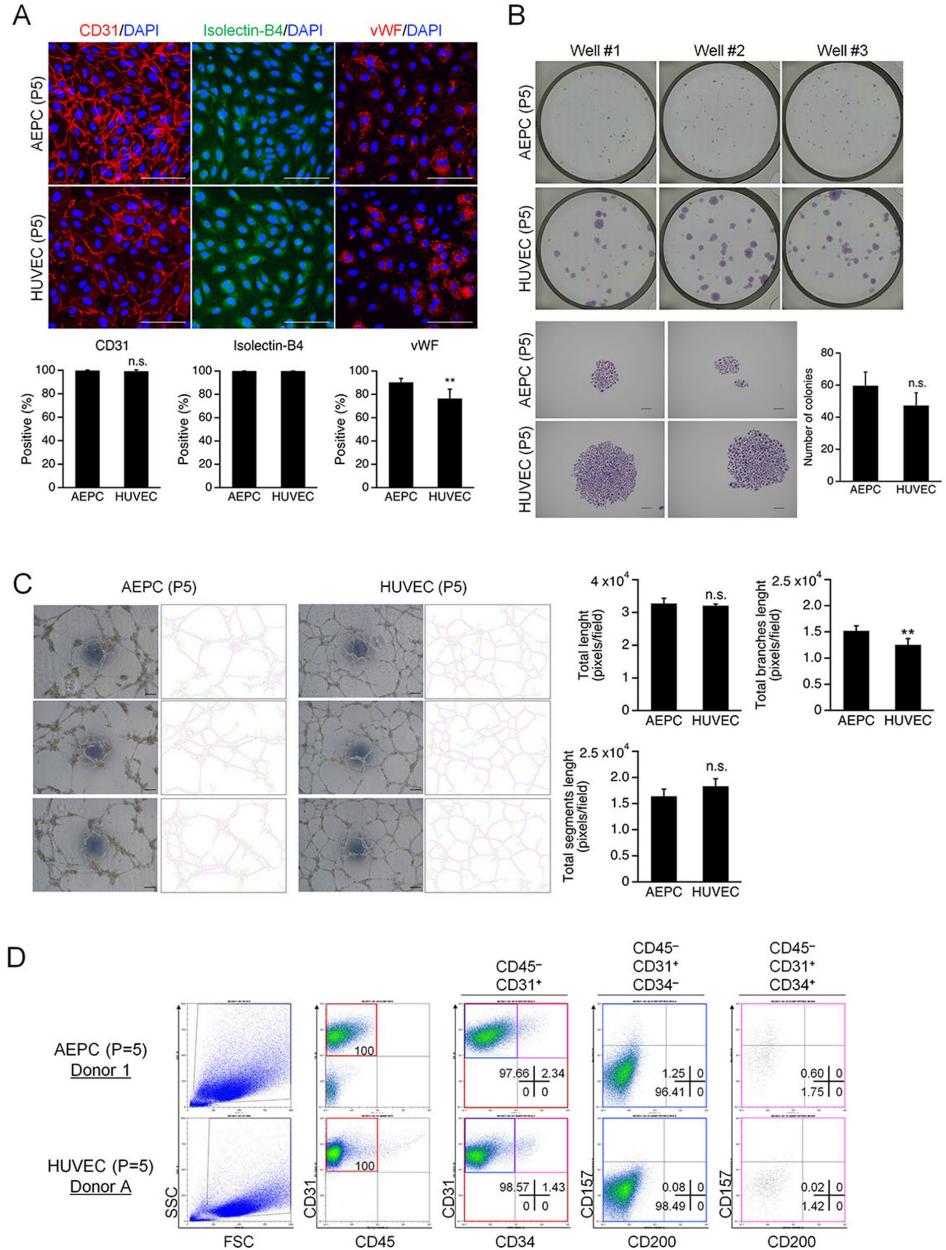
Characterization and functional analysis of expanded AEPCs compared with HUVECs. All the experiments were performed using AEPCs and HUVECs at passage number 5. (**A**) Fluorescent immunocytochemistry of the expression profiles of endothelial cell-specific markers, CD31, vWF, and the binding capacity of isolectin-B4, in AEPCs and HUVECs. Nuclear staining was assessed by 4′,6-diamidino-2-phenylindole (DAPI). Photographs were taken using a confocal microscope (OLYMPUS, FV1000 with a CCD camera DP71 and an objective lens UPlanFL N 40×/1.30 oil; 400× magnification) as a plane image. Bars represent 100 µm. The graphs show the percentage of endothelial marker-positive cells among DAPI-positive cells. The data represent two independent experiments (2 donors), and percentages were determined from cultivations performed in triplicate. The bars represent means ± SD (n = 6, **P < 0.01, two-tailed unpaired t-test). (**B**) CFU-EC analysis of AEPCs and HUVECs. Cells were seeded at 125 cells per 35-mm-diameter well (12.6 cells/cm^2^) and cultured for 7 days. Each experiment was performed twice independently (two donors) in technical triplicates. The bars represent means ± SD (n = 3; n.s., not significant by two-tailed unpaired t-test). (**C**) Network formation capacity of AEPCs compared with that of HUVECs. Representative phase-contrast pictures corresponding to the right-hand side of the extracted network skeleton. The total segment length (magenta color), total branch length (yellow–green color), and total length were determined using the Angiogenesis Analyzer plugin for ImageJ software. Scale bars represent 100 µm. The experiment was performed independently three times (3 donors) in technical pentaplicates. Bars represent means ± SD (n = 5, **P < 0.01; n.s., not significant by two-tailed unpaired t-test). (**D**) Cultured AEPCs and HUVECs were characterized by the expression of CD45, CD31, CD34, CD157, and CD200 by flow cytometry analysis. This experiment was performed independently three times (3 donors) for AEPCs and independently two times (2 lots, 5- or 7-donor mixture) for HUVECs and is listed in Table 2.

We characterized and performed functional analyses of expanded AEPCs (passage 5), compared against expanded HUVECs (passage 5), using immunocytochemistry, CFU-EC, EC network formation, and flow cytometry analyses. Immunocytochemistry showed similar CD31 and vWF expression profiles and isolectin-B4 binding capacity between AEPCs and HUVECs (Fig. 5A). CD31 expression was detected in 99.8 ± 0.4% and 99.3 ± 1.1% of AEPCs and HUVECs, respectively. vWF was expressed in 90.3 ± 3.3% and 76.4 ± 8.0% of AEPCs and HUVECs, respectively. Both AEPCs and HUVECs were 100% positive for isolectin-B4 binding capacity.

In the CFU-EC assay, the colony forming cells in AEPCs and HUVECs were 59.7 ± 8.4% and 47.3 ± 7.9%, respectively (which did not differ significantly) on day 7. AEPCs proliferated more slowly in colonies, and the average size of AEPC colonies was smaller than that of HUVEC colonies (Fig. 5B). Even if using the same medium, EGM-2MV, AEPCs formed colonies smaller in size than HUVECs, and reached a similar size on day 12 to that of HUVECs on day 7 (Supplemental Figure S3).

In the network formation assay, both AEPCs and HUVECs formed tube-like networks. The total length, total segment length, and total branch length were analyzed, and all the parameters were similar between AEPCs and HUVECs. The total network lengths measured for AEPCs and HUVECs were 32.7 ± 1.8 × 10^3^ and 32.1 ± 0.5 × 10^3^ pixels/field, respectively. The total segment lengths for AEPCs and HUVECs were 16.4 ± 1.5 × 10^3^ and 18.3 ± 1.6 × 10^3^ pixels/field, respectively. The total branch lengths for AEPCs and HUVECs were 15.2 ± 1.1 × 10^3^ and 12.5 ± 1.3 × 10^3^ pixels/field, respectively (Fig. 5C).

Flow cytometry indicated that expanded AEPCs expressed reduced levels of tissue-resident EPC markers, such as CD157 and CD200, during culture, and CD200 was not detectable by passage number 5. AEPCs (passage 5) contained CD157^+^ population at 1.1–2.7% among CD45^−^CD31^+^ cells (n = 3). By contrast, HUVECs (passage 5) did not express either CD157 or CD200 (Fig. 5D and Table 2).

**Table 2:** List of the expression profiles of cultured AEPCs and HUVECs.

Donor	Cell type	Donor information	CD markers					Population (%)	CD45^−^CD31^+^CD157^+^CD200^−^ population (%)
			CD 45	CD 31	CD 157	CD 200	CD 34		
1	AEPCs (*P* = 5)	Aged 45 years, Female	−	+	+	−	−	1.25	1.85
							+	0.60	
2	AEPCs (*P* = 6)	Aged 57 years, Female					−	2.39	2.69
							+	0.30	
3	AEPCs (*P* = 5)	Aged 28 years, Male					−	0.94	1.13
							+	0.19	
A	HUVECs (*P* = 5)	5-donor mixture					−	0.08	0.10
							+	0.02	
B	HUVECs (*P* = 5)	7-donor mixture					−	0.12	0.12
							+	0.00	

## Discussion

Although human microvascular AEPCs are an important cell population, two primary challenges have prevented the establishment of purification and expansion methods. First, AEPCs are easily overwhelmed by contaminating ASCs during the adherent culture process. Second, AEPCs appear to be more vulnerable to tissue enzymatic dissociation processes than ASCs. Both the total nucleated SVF yield and ASC yield increased with increasing collagenase concentrations between 0.02 and 2% (w/v), whereas the optimal AEPC yield was observed at collagenase concentrations between 0.1 and 0.4%. In addition, the SVF obtained from adipose tissue showed differential population profiles depending on the supplemental reagents used during extraction. The addition of a biocompatible amphipathic detergent, Pol188, increased the extraction efficiency of total SVF and ASCs from adipose tissue^24^; however, the extraction efficiency of AEPCs decreased following the addition of Pol188. AEPCs and ASCs both localize in proximity to the microvasculature of adipose tissue^26,27^ and are exposed to the enzymatic dissociation process in a similar manner, suggesting that AEPCs are more sensitive to artificial stimuli.

Instead of FACS purification, MACS separation was used in this study because the ultimate goal is the clinical application of AEPCs that requires a sufficiently large number of viable cells. MACS separation can process a much larger number of cells within a shorter time without damaging their viability although its purification efficiency is inferior to that of FACS. Because of the same reason, this roughly separated population was assessed instead of AEPCs collected from colony formation.

During the flow cytometric analysis, the populations of freshly isolated AEPCs and ASCs were characterized as CD45^−^CD31^+^CD34^+^CD105^+^CD146^+^CD157^±^CD200^±^ and CD45^−^CD31^−^CD34^+^CD105^−^CD146^−^CD157^±^CD200^−^ (Fig. 1B), respectively, a finding that is partly supported by that in a previous study^28^. CD34 expression gradually decreased in AEPCs over the culture period, whereas CD34 expression was drastically reduced in ASCs as early as passage 2 (Fig. 4). The purification of AEPCs was completed when the CD31-positive population was at 97%, but not at 94% (Fig. 3C). This difference was small but changed the final result of purification. AEPCs were successfully purified using MACS separation and could be successfully expanded through passage 5, although they continued to be contaminated with a small number of ASCs (approximately 3%), suggesting that the purification rate of AEPCs is a critical factor to avoid ASC expansion. AEPCs at passage 5 showed reduced CD31 expression (< 80%) by flow cytometry, and other morphological cell types could not be observed under the microscope, as confirmed by the immunocytochemical observation of CD31 staining (Figs. 4A and 5A). Some studies have reported that ECs show decreased CD31/PECAM-1 expression in a time-dependent manner in vitro^29,30^, and AEPCs may also decrease CD31 protein expression following extended expansion. In the CFU-EC assay, AEPC colonies proliferated more slowly than HUVECs (Figs. 5B and S3), exhibiting smaller colonies in size than HUVECs.

EPCs are difficult to obtain from circulating peripheral blood (CD34^+^CD133^+^VEGFR-2^+^ expressed cells), with one report identifying only 1074 EPCs in 20 mL of peripheral blood^31^. In addition, bone marrow-derived EPCs have a controversial issue. Although some papers have reported that bone marrow-derived EPCs are mobilized to undergo temporarily angiogenesis in the inflammatory phase and granulation tissue construction phase of the wound healing process, others have indicated that not circulating EPCs but tissue resident EPCs, such as AEPCs, predominantly contributed to neovascularization^32–34^. Considering clinical applications, it is more reasonable to purify and expand AEPCs from the microvasculature of human adipose tissue^23,35^. One research group successfully purified AEPCs from large-volume human adipose tissue using Dynabeads magnetic sorting combining the CD44^−^ and CD90^−^ fractions. However, they reported that “Adipose tissue ECs may be a more practical alternative for obtaining large quantities of autologous ECs”^36^. However, our method has resolved the issue. CD157, a novel tissue-resident EPC marker, has previously been detected in the portal vein and adipose tissue blood vessels of mice^9,11^. Three populations of VE-cadherin^+^CD31^+^CD45^−^ EPCs were reported: CD157^+^CD200^+^, CD157^−^CD200^+^, and CD157^−^CD200^−^. In addition, CD157^+^ cells were recognized to generate functional blood vessels in a mouse liver injury model^11^. In our study, expanded AEPCs contained CD157^+^ cells through at least passage number 5, although the percentage was small. In addition, expanded AEPCs exhibited clonal expansion capacity, self-renewal capacity, EC-specific characteristics, such as vWF expression, lectin binding, and network formation capacity, indicated that AEPCs feature progenitor characteristics.

## Conclusions

Our study is the first to characterize human AEPCs and establish a purification and a large-scale (clinical scale) expansion method to isolate these cells. Because adipose tissue is a clinically accessible and abundant tissue, AEPCs have potential advantages as a therapeutic tool for regenerative medicine and surgical applications compared with EPCs derived from other sources, such as bone marrow and peripheral blood. The preclinical and clinical value of AEPCs to treat potential target diseases, such as ischemic pathologies, remains to be established in future studies.

### Patent information

The preparation method used for adipose-derived endothelial (progenitor) cells and its use as a medical formulation are patent pending (Japanese Patent Application Disclosure, P2019-88279A). The medical-grade enzymatic formulation used to extract SVF to effectively purify both adipose-derived endothelial (progenitor) cells and adipose-derived stem cells using GMP-grade enzymes is patent pending (Patent Application Disclosure, WO2021-112168).

## Supplementary Information

Supplementary Information.

## Data Availability

The datasets are available from the corresponding author upon reasonable request. Ethical restriction exists on sharing the original study datasets.

## Abbreviations

EPCs: Endothelial progenitor cells
ECs: Endothelial cells
IRB: Institutional review board
SVF: Stromal vascular fraction
AEPCs: Adipose-resident microvascular endothelial progenitor cells
DNase1: Deoxyribonuclease 1
Pol188: Poloxamer 188
HBSS: Hanks’ balanced salt solution
MACS: Magnetic-activated cell sorting
FBS: Fetal bovine serum
FVD780: Fixable viability dye eFluor 780
HUVECs: Human umbilical vein endothelial cells
FACS: Fluorescence-activated cell sorting
BSA: Bovine serum albumin
EDTA: Ethylenediamine-*N*,*N*,*N*′,*N*′-tetraacetic acid
DPBS: Dulbecco’s phosphate-buffered saline
RT: Room temperature
vWF: Von Willebrand factor
DAPI: 4′,6-Diamidino-2-phenylindole
PFA: Paraformaldehyde in phosphate buffer solution
PBS: Phosphate-buffered saline
CFU-EC: EC colony-forming unit
ASCs: Adipose-derived stem cells
SD: Standard deviation
GMP: Good manufacturing practice
