# Supplementary material for: Purification and characterization of human adipose-resident microvascular endothelial progenitor cells
Source: Sci Rep. 2022 Feb 2;12:1775. doi: 10.1038/s41598-022-05760-4 (PMC8811023; doi:10.1038/s41598-022-05760-4)
Supplement: Supplementary file 1 — Supplementary Information. [file 41598_2022_5760_MOESM1_ESM.docx]

**Supplemental Material**

**Title:**

Purification and characterization of human adipose-resident microvascular endothelial progenitor cells

| Component | EGM-2 | EGM-2MV |
| --- | --- | --- |
| EBM-2 Endothelial Cell Basal Medium-2 * | 250 mL | |
| hEGF ** | 0.25 mL | |
| VEGF ** | 0.25 mL | |
| R3-IGF-1 ** | 0.25 mL | |
| Ascorbic Acid ** | 0.25 mL | |
| Hydrocortisone ** | 0.1 mL | |
| hFGF-b ** | 1.0 mL | |
| GA-1000 ** | 0.25 mL | |
| Heparin ** | 0.25 mL | – |
| FBS (Sigma-Aldrich, #F7524) | 5.0 mL | 12.5 mL |
| * Basal media was used by half-and-half of one bottle or a single lot of the media. | | |
| ** Growth factors were added from a single lot of EGM-2 SingleQuots Supplements. | | |

**Supplemental Table S1. Preparation of EGM-2 and EGM-2MV media in this study**

**
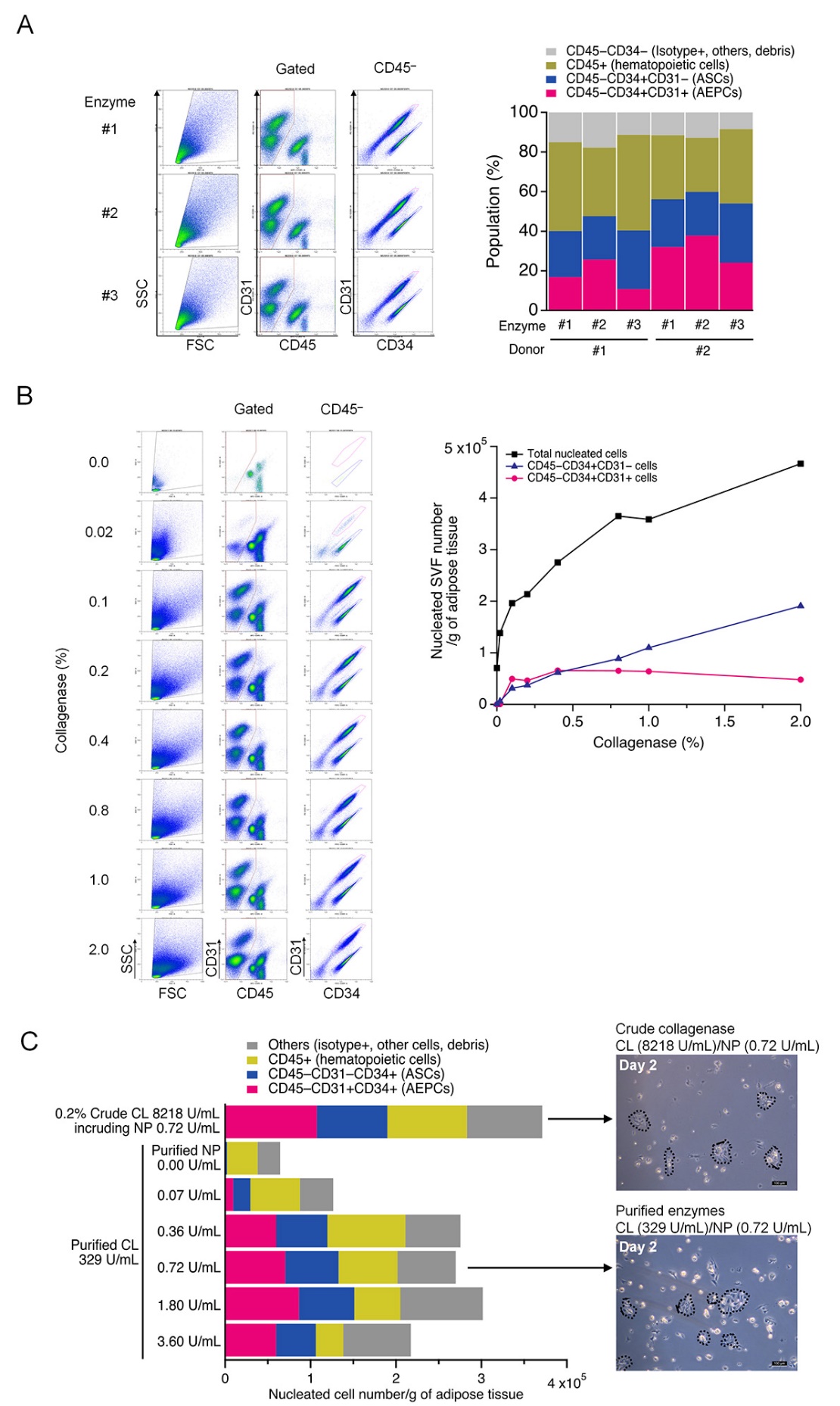
**

**Supplemental Fig. S1**. **Preliminary experiments to optimize adipose digestion processes for isolation of AEPCs**

**(A)** SVF was extracted using three types of enzyme mixtures to determine the optimal enzyme composition for AEPC purification. (1) Collagenase and CaCl_2_ in HBSS. (2) Collagenase, CaCl_2_, and DNase1 in HBSS. (3) Collagenase, CaCl_2_, DNase1, and Pol188 in HBSS. Each SVF was examined for CD45, CD34, and CD31 expression profiles using flow cytometry. In this analysis, the lymphocyte-gated population was set to 100%. The population consists of a CD45^−^CD34^+^CD31^+^ AEPC population (magenta), a CD45^−^CD34^+^CD31^−^ ASC population (blue), a CD45^+^ hematopoietic cell population (olive), and a population containing CD45^−^CD34^−^ isotype+ cells, other cells, and debris (gray). **(B)** Optimization of the collagenase concentration for AEPC purification. The displayed collagenase concentration indicates the prepared collagenase value. The enzymatic reaction was performed by combining the prepared collagenase with adipose tissue at 1:1 so that the final collagenase values during the enzymatic reaction were half the listed concentrations. The experiment was performed independently three times, using 3 independent donors. The results of all independent experiments showed the same tendencies. **(C)** Assessment of 0.2% (w/v) collagenase (crude type; FUJIFILM Wako, #032-22364) for total protein (Thermo Fisher Scientific, Pierce BCA Protein Assay Kit, #23225), collagenase activity (Thermo Fisher Scientific, EnzChek Gelatinase/Collagenase Assay Kit, #E12055), and neutral protease activity with the FAGFYA method ^1^; 0.2% (w/v) crude collagenase contained 8218 units (U)/mL of collagenase (CL) activity and 0.72 U/mL of neutral protease (NP) activity. SVF was extracted using 329 U/mL of purified CL (Amano Enzyme, #Collagenase “Amano” GMP) with purified NP (Amano Enzyme, #Clostlysin “Amano” GMP) at 0, 0.07, 0.36, 0.72, 1.80, and 3.60 U/mL. The photos were obtained on day 2 in culture with a phase-contrast microscope (Leica, DM IL LED with a camera MC170HD). Dotted circles indicate AEPC colonies. Bars represent 100 μm.


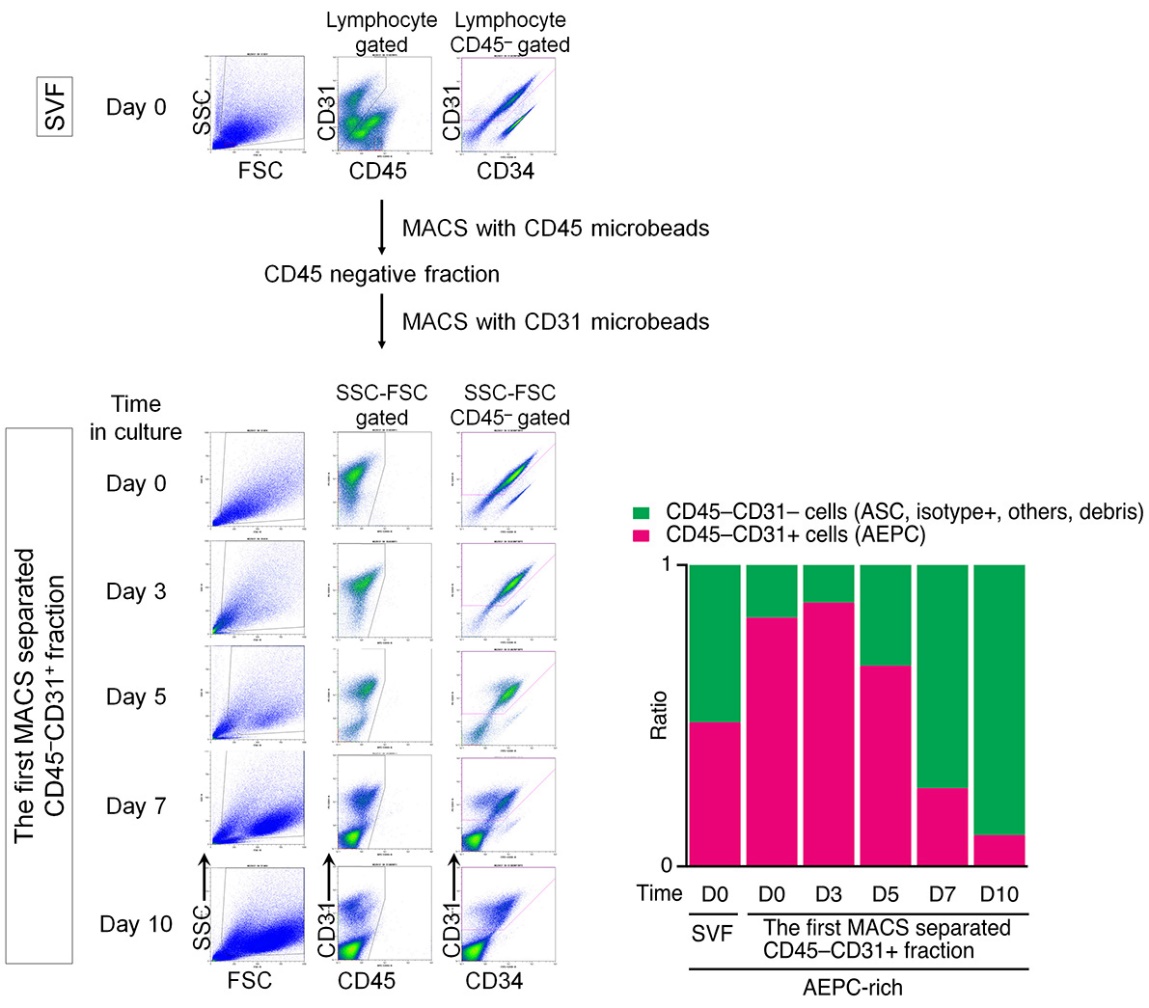


**Supplemental Fig. S2**. **Optimization of the culture period after the first MACS separation**

The first MACS-separated CD45^−^CD31^+^ (AEPC-rich) fraction was cultured in EGM-2MV media for 10 days. The cells were analyzed for CD45, CD34, and CD31 expression. The CD45^−^CD31^−^ population, representing proliferating ASCs, increased after day 7 in culture. The ratio of CD45^−^CD31^+^ (AEPCs) to CD45^−^CD31^−^ (ASCs, other cells, isotype^+^ cells, and debris) populations on days 0, 3, 5, 7, and 10 in culture were 0.83, 0.88, 0.67, 0.26, and 0.10, respectively, (n = 1). Therefore, we attempted the second MACS separation on days 4.5 and 7, as shown in Fig. 3.


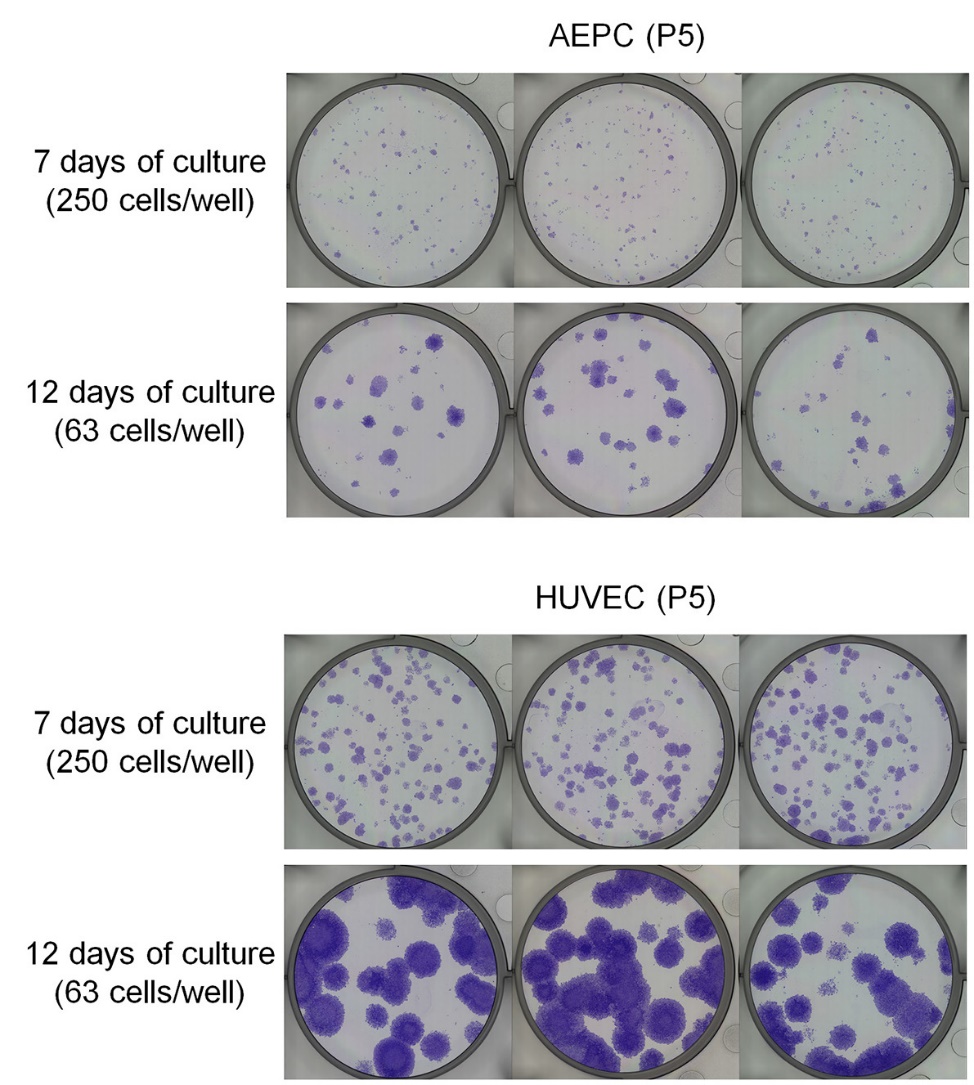


**Supplemental Fig. S3** CFU-EC assay of AEPCs and HUVECs in EGM-2MV medium.

Cells were seeded on cell culture surface at either 250 cells or 63 cells per 35-mm-diameter well (25.5 or 6.4 cells/cm^2^), and these cells cultured for either 7 days or 12 days, respectively.

**Abbreviations**

Collagenase, CL; neutral protease, NP.

**Reference**

1 Maeda, H. *et al.* Cloning a neutral protease of Clostridium histolyticum, determining its substrate specificity, and designing a specific substrate. *Appl Microbiol Biotechnol* **99**, 10489-10499, doi:10.1007/s00253-015-6923-4 (2015).
